# The Difference of Brain Functional Connectivity between Eyes-Closed and Eyes-Open Using Graph Theoretical Analysis

**DOI:** 10.1155/2013/976365

**Published:** 2013-04-17

**Authors:** Bo Tan, Xianxian Kong, Ping Yang, Zhenlan Jin, Ling Li

**Affiliations:** Key Laboratory for NeuroInformation of Ministry of Education, School of Life Science and Technology, University of Electronic Science and Technology of China, Chengdu 610054, China

## Abstract

To study the differences in functional brain networks between eyes-closed (EC) and eyes-open (EO) at resting state, electroencephalographic (EEG) activity was recorded in 21 normal adults during EC and EO states. The synchronization likelihood (SL) was applied to measure correlations between all pairwise EEG channels, and then the SL matrices were converted to graphs by thresholding. Graphs were measured by topological parameters in theta (4–7 Hz), alpha (8–13 Hz), and beta (14–30 Hz) bands. By changing from EC to EO states, mean cluster coefficients decreased in both theta and alpha bands, but mean shortest path lengths became shorter only in the alpha band. In addition, local efficiencies decreased in both theta and alpha bands, while global efficiencies in the alpha band increased inversely. Opening the eyes decreased both nodes and connections in frontal area in the theta band, and also decreased those in bilateral posterior areas in the alpha band. These results suggested that a combination of the SL and graph theory methods may be a useful tool for distinguishing states of EC and EO. The differences in functional connectivity between EC and EO states may reflect the difference of information communication in human brain.

## 1. Introduction

The human brain is one of the most complex systems and many neurophysiological mechanisms are still unclear. Since Hans Berger first recorded EEG signals and then published the first paper about scalp EEG, EEG was widely applied to the research of the human brain [1]. Brain functions, such as attention, learning, emotion, and working memory, can be reflected by EEG dynamics [2, 3]. To further explore the human brain, brain connectivity has become a popular research field over the past 10 years. Interactions among different regions of the human brain were defined as brain connectivity, mainly including effective connectivity, functional connectivity, and anatomical connectivity [4, 5]. The aim of this paper is to find out the differences in functional brain networks between EC and EO at resting state.

Many previous studies showed that the brain was in a “default mode” without any external task at eyes-closed resting state and the power of the theta significantly decreased in frontal area from EC to EO states which were based upon EEG and functional magnetic resonance imaging (fMRI) studies [3, 6–8]. Meanwhile, the recent EEG studies also showed that the power of the alpha band was distributed in bilateral posterior area with significant reduction when subjects had their eyes open [6, 9, 10]. However, in the past dozens of years, EEG and fMRI studies of the differences between EC and EO states were mainly based on the power spectrum [6, 7], and few studies showed the difference in functional brain networks between EC and EO states.

Some different measurements could be applied to estimate the functional connectivity between all pairwise channels, mostly including linear temporal correlation, phase synchronization, and generalized synchronization [11–13]. However, linear temporal correlation is insensitive to asymmetric or nonlinear interdependencies and cannot be well used to analyze nonstationary signal (e.g., EEG signal) [11, 12]. The phase synchronization is usually applied in evoked EEG analysis based on single-trial [13]. Recently, according to the theory of generalized synchronization, several algorithms such as mutual false nearest neighbors (MFNNs), nearest neighbors, and mutual nearest neighbors have been introduced to detect this type of interdependencies in experimental time series and overcome some of the limitations of the above methods [14, 15]. However, these algorithms have bias when relating to the degrees of freedom of the interacting subsystems. In order to deal with nonstationary dynamics and avoid this bias, Stam and Dijk proposed synchronization likelihood (SL) to measure statistical dependencies in a dynamical system [11, 14]. As the SL was suitable to analyze nonstationary signal by calculating in a time-dependent way, it has been proved to be very useful to study interactions among neurons and measure brain functional connectivity in multivariate data [11, 14, 15]. In addition, the SL was often used for studying neurological disorder during a working memory task or a no-task state [13–15]. Moreover, some important applications of the SL were about epilepsy and gamma band synchronization in magneto encephalography (MEG) data [11]. Meanwhile, the measurement of SL was also helpful to study task-dependent changes in healthy people during eyes-closed or eyes-open state in EEG data [11]. However, previous studies concentrated on the comparison of the synchronization between EC and EO states only in the alpha, they did not further analyze the difference in functional brain networks which were constructed by the SL. In this paper, the SL was to measure correlations of EC and EO states between all pairwise EEG channels.

Graph theory is popularly applied to study property of brain networks and compare the differences among complex networks [16, 17]. Usually, the brain was expressed as a network graph which consisted of nodes and edges [5, 16]. In our EEG analysis, the nodes typically correspond to separate channels and the edges exist when the value of synchronization exceeded corresponding threshold among all pairwise EEG channels. A network graph was usually characterized by some topological parameters, such as the clustering coefficient, the shortest path length, the global efficiency, and the local efficiency [17–19]. Meanwhile, ever since Watts and Strogatz found that small-world networks had the feature of a high-clustering coefficient and a short path length [19, 20], many networks in reality have been proved to have small-world features, including the human brain. Barahona and Pecora also pointed out that the synchronous neural activities among different brain regions conform to small-world network architecture [21]. Our results further supported that functional brain networks have small-world features at EC and EO states.

In this paper, in order to compare functional brain networks of EC and EO states, two sessions were designed as an experiment, including three minutes of eyes-closed resting state and three minutes of eyes-open resting state with binocular fixating a center green across [6, 7]. The EEG data was filtered into the following three separate frequency bands: theta (4–7 Hz), alpha (8–13 Hz), and beta (14–30 Hz) bands, and the same analysis process was used in each frequency band. The SL matrices were calculated and then converted to graphs. Topological parameters as well as the difference connections (nodes) between EC and EO states were analyzed [16, 17]. The difference nodes of networks were examined by the crosstab test and the chi-square test in the entire thresholds. Besides, difference connections were obtained by the chi-square test for each threshold. The above methods and techniques were used to address the following hypotheses. (a) The topological parameters should have significant changes from EC to EO states in the theta band, since the power of the theta band in the frontal area decreased from EC to EO state [6, 8, 9]. (b) The change of graph measures should be more obvious and the local brain connections should reduce in bilateral posterior area in the alpha band from EC to EO states, in consideration of the human alpha rhythms which are dominant in most of normal adults and exhibited bilateral distribution over the posterior areas in EC state [9, 22]. Combination of the SL and graph theory methods in the present study would be a very useful tool for exploring underlying mechanism of the brain and for diagnosing neurologic disorder in the future.

## 2. Methods

### 2.1. Participants and Experiment

Twenty-one healthy right-handed subjects (ten females and eleven males) between 20 and 23 years old (Mean_age_ = 21.6 years, SD = 0.8) from the university community participated in the present study. All participants were free of personal or family history of neurological disorder or serious mental illness and had no history of substance abuse or head injury. In addition, they were not taking any psychoactive medications. All participants had no visual or auditory impairments. Written informed consent was given by all participants before the EEG experiment, and they were compensated for their participation.

The experiment included two processes: eyes-closed and eyes-open at resting state [6, 7]. During the experiment, subjects were comfortably seated in a dimly lit, sound attenuated, and electrically shielded environment. First, each subject kept eyes-closed state for three minutes without thinking of anything while in a fully relaxed state. Second, the subjects needed to keep their eyes-open for another 3 minutes while focusing on a green cross in the middle of screen without any instruction and staying in a fully relaxed state.

### 2.2. EEG Data Acquisitions and Preprocessing

EEG data were recorded from 128 channels by using a modified 10–20 montage system (EGI system) in all subjects. The reference electrode was the Cz (129th) electrode, and electrooculogram (EOG) was recorded simultaneously from electrodes placed above and below the left eye. EEG data were sampled at 500 Hz and the electrode impedance was kept lower than 5 kΩ. EEG data during three minutes eyes-closed state and three minutes eyes-open state were collected for further analysis. All the EEG data were referenced to an averaged reference by offline way.

Bad electrodes were replaced with the interpolated values from the neighboring electrodes. After rejecting EOG contamination and nonspecific artifacts, 5000 time points (ten seconds EEG data) were selected from the original time series of each subject. The ten seconds EEG data were filtered into the following three distinct frequency bands by a band-pass filter: theta (4–7 Hz), alpha (8–13 Hz), and beta (14–30 Hz) bands. In addition, the filtered EEG data were processed by the current source density (CSD) toolbox of Matlab v7.1 (Mathworks, Inc., Sherborn, MA) which was supplied by Kayser and Tenke which implements a spherical spline algorithm of Perrin et al. to estimate scalp current density (SCD) [23, 24].

### 2.3. Functional Brain Network Construction

#### 2.3.1. Synchronization Likelihood (SL)

For two dynamical systems *X* and *Y*, when *Y* is determined by a function *F* which is driven by *X*, which is called generalized synchronization between system *X* and *Y*. Stam and Van Dijk proposed a measurement of synchronization likelihood (SL) to estimate generalized synchronization between two or more simultaneously recorded time series [11, 14]. Because the SL is sensitive to linear and nonlinear mutual dependencies, it can be well applied to measure the correlations between any two EEG channels [14, 15]. 

Computing the SL of simultaneously recorded time series *X* and *Y*, the first step is to reconstruct the series embedded vectors *X*_*i*_ and *Y*_*i*_ by the method of time-delay embedding [11, 14, 15]:

(1) $$\begin{array}{c} X_{i}=\left(x_{i},x_{i+l},x_{i+2l},\ldots ,x_{i+(m-l)l}\right), \end{array}$$

where *i* denotes discrete time, *l* is the time delay, and *m* is the embedding dimension. The *Y*_*i*_ is constructed by the same way.

The second step is to define the probability *p*(*r*_*i*_^*x*^) for each time *i* of the time series *X*. The cutoff distance *r*_*i*_^*x*^ can be determined by *p*(*r*_*i*_^*x*^) = *p*_ref_, where *p*_ref_ does not depend on the properties of the time series and can be set at an arbitrarily low level and far less than 1:

(2) $$\begin{array}{c} p\left(r_{i}^{x}\right)=\frac{1}{2\left(w_{2}-w_{1}\right)}\sum_{\begin{array}{c} w_{1}<|i-j|<w_{2}\\ j=1 \end{array}}^{N}\theta \left(r_{i}^{x}-\left|X_{i}-X_{j}\right|\right), \end{array}$$

where *θ* is the Heaviside step function (*θ*(*x*) = 1 for *x* > 0 and *θ*(*x*) = 0 for *x* ≤ 0), and the |·| is the Euclidean distance between *X*_*i*_ and *X*_*j*_. *w*_1_ is a window to correct autocorrelation effects by the Theiler and should be more than the order number of the autocorrelation time, *w*_2_ is a window to improve the time resolution of the synchronization measure, and the relationship must be met: *w*_1_ ≪ *w*_2_ ≪ *N*, *N* is the number of discrete time points. The cutoff distance *r*_*i*_^*y*^ of each time can be calculated through the same process [11, 14, 15].

The next step is to define the SL of the discrete time *i*; however, the SL_*i*_ is related with the value of |*X*_*i*_ − *X*_*j*_|, and if the |*X*_*i*_ − *X*_*j*_ | ≥*r*_*i*_^*x*^, the SL_*i*_ was zero; if |*X*_*i*_ − *X*_*j*_ | <*r*_*i*_^*x*^, we can calculate the SL_*i*_ of the discrete time *i*. Now the SL_*i*_ of the discrete time *i* can be formally defined as follows [11]:

(3) if |Xi−Xj|≥rix:SLi=0if |Xi−Xj|<rix:SLi=12(w2−w1)×pref     ×∑w1<|i−j|<w2j=1Nθ(riy−|Yi−Yj|).

The values of SL range from *p*_ref_ to 1. In case of maximal synchronization, the SL of the time *i* is 1. If the two systems are completely independent systems, the value of SL is *p*_ref_ [11, 15]. 

Finally, the SL between the time series *X* and *Y*can be obtained by averaging over the time index *i*. Otherwise, the definition of SL is closely related with the concept of mutual information which is based on the correlation integral [11, 15]. The SL is sensitive to linear and nonlinear systems; so it has been applied to explore human multichannel EEG and MEG data, including neurological disorders (e.g., Epilepsy, Alzheimer's disease) [14, 15] and advanced brain functions (e.g., attention, perception).

According to the fixed parameter selection rules in the definition of SL and previous experience of the applications in EEG literatures [11, 14], the following fixed embedding parameters were selected to estimate SL value in this study: lag (*l*) is 10; embedding dimension (*m*) is 10; two windows: *w*_1_ is 100 and *w*_2_ is 400; and *p*_ref_ was set equal to 0.05.

For each subject, an SL value was obtained for one pair of channels in each frequency band. After computing SL values of all pairwise combinations of channels, all SL values constituted a square *M* × *M* synchronism matrix of size 128 (128 is the number of EEG channels), where each element *M*_*i*,*j*_ is the value of the SL between channel *i* and *j*. Finally, the number of SL matrixes is 126 (21 × 3 × 2, 21 subjects, 3 frequency bands, and 2 conditions).

#### 2.3.2. Threshold Selection

The threshold selection was based on the cost of functional brain network [16, 25], which was defined as the ratio of the number of above-threshold edges to the total number of all edges in a network. For example, when the threshold was at a cost of 20%, it means that only 20% of the top SL values were considered as effective connections and the other 80% SL values were considered as invalid connections. 

**Figure 1:**
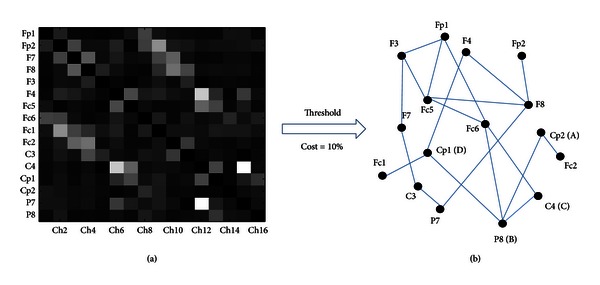
The schematic diagram that an SL matrix was converted to an undirected and unweighted graph by applying a threshold. (a) is an SL matrix and (b) is an undirected and unweighted graph. In case of an unweighted and undirected graph, the black solid dots represent nodes and would be connected by edges when SL exceeded the corresponding threshold. Topological features of graphs can be quantitatively described by a wide variety of measures. For node D, which links three edges, the degree is three, and all the networks' nodes form a degree distribution. For node A, with neighbors B and C, the clustering coefficient is one. The shortest path between node A and node D consists of three edges, and the shortest path length is three. Otherwise, if one node cannot reach another node, the distance is considered as infinite.

In this study, each network was examined in the full range of costs from 8% to 20% (step is 1%). The 8% was based on keeping small-world feature of functional brain network, while the network connection was too dense to further analyze network topology when the cost exceeded 20% [20, 25]. After applying each threshold (13 thresholds), the SL matrices were converted to undirected connected graphs. An edge was set when the SL value exceeded corresponding threshold in Figure 1. Finally, the number of all graphs is 1638 (21 × 3 × 2 × 13, 21 subjects, 3 frequency bands, 2 conditions, and 13 thresholds). 

### 2.4. Graph Theory Analysis

#### 2.4.1. Network Measures

After the SL matrices were converted to graphs consisting of nodes and undirected edges between nodes in Figure 1 [5, 26], several regular graph-theoretic parameters were selected to analyze functional brain networks of EC and EO states, including the average degree *K*_*m*_, the clustering coefficient *C*, the mean shortest path length *L*, the global efficiency *E*_global_, and the average local efficiency *E*_local_ [17–19, 25]. Mathematical details of these parameters can be found in the appendix of this paper.

The average degree, clustering coefficient, and mean path length are basic and core measures of graphs. The average degree could reflect the mean density of connections among nodes in a graph. Due to the threshold selection based on the costs of networks, the difference of the degree between EC and EO states is not significant. The clustering coefficient is often used for measuring local structure and has been regarded as an index of resilience to random error in a graph [17, 18]. The mean path length is a global characteristic and can reflect the integration as well as the level of information communication in a graph [18, 19]. In addition, the global and local efficiency can reflect the level of global and local information transfer of a graph and are directly and effectively applied to evaluate the performance of a network [19, 25]. 

#### 2.4.2. Difference Nodes and Difference Connections

Many previous studies have reported that there existed some nodes with a high degree or high centrality in a graph [26–28]. The centrality of a graph was used to measure how many of the shortest paths between all other node pairs in the network pass through it, and it would have a great influence on network efficiency [19, 29, 30]. To further study the difference of network topology between the two states, the crosstab test was used to detect the difference nodes of EC and EO states in this study. A node was determined as a difference node of EC state when the degree of the node in EC state was always significantly greater than that of EO state at each cost. On the contrary, when the degree of a node of EO state was always significantly greater than that of EC state at each cost, the node was considered as a difference node of EO state. 

In order to further compare the difference of network connections, we constructed the connected graph of two states and the difference connected graph between the two states. The connected graph was composed of the edges which were significant among all subjects by the chi-square test. Then we can, respectively, obtain a connected graph of EC state and a connected-graph of EO state at each cost. However, the difference connected-graph was constructed by comparing the connected-graphs of EC and EO states, if the same connection exits between two nodes of EC and EO states in connected-graphs, the connections would be ignored and would not be drawn in the difference connected-graph. If only EC state had an edge in connected graph, the two nodes would be linked by blue line. Otherwise, the two nodes were linked by red line. Finally, difference connected-graphs were acquired in each frequency band for each subject at each cost.

### 2.5. Statistical Analysis

Paired *t*-test was applied to evaluate the differences in topological parameters and the connectivity of networks between EC and EO states. The chi-square test and the crosstab test were used to estimate the significant difference of nodes and connections between EC and EO states. *P* < 0.05 was accepted as significant in all tests. All operations were done in Matrix Laboratory (Matlab v7.1) (Mathworks, In.c, Sherborn, MA). The statistical procedures were completed in Statistical Product and Service Solutions (SPSS v20.0) (SPSS, Chicago, IL, USA).

## 3. Results 

After SL matrices were converted to graphs, there were network analysis and statistical analysis in each frequency band for each subject separately. Some significant differences of network parameters, difference nodes, and difference connections were found between EC and EO resting conditions.

### 3.1. The Theta Band

#### 3.1.1. Network Parameters

**Figure 2:**
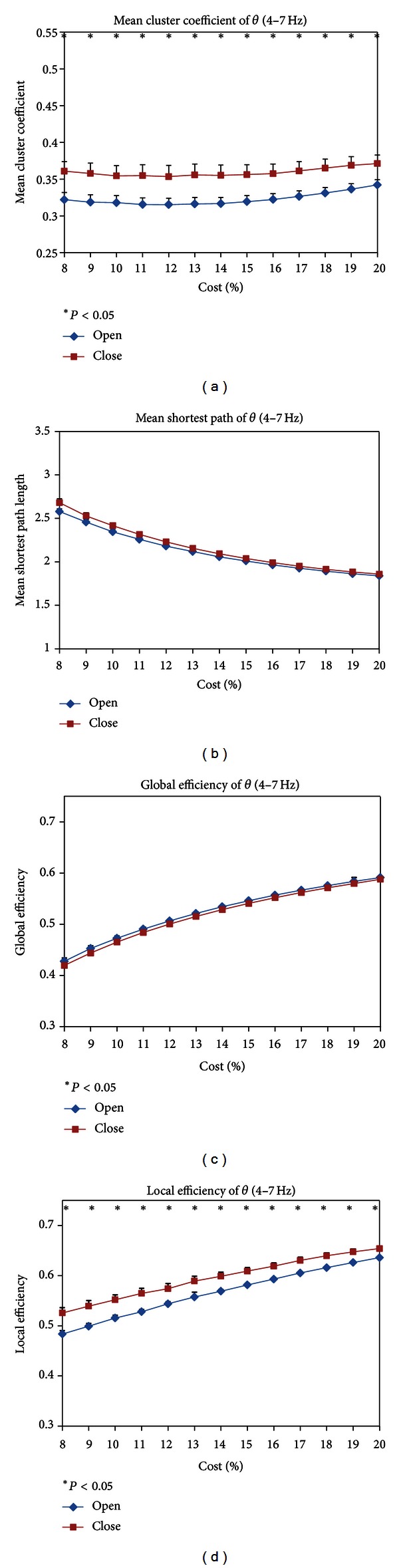
(a) mean cluster coefficient *C*_*s*_, (b) mean shortest path length *L*_*s*_, (c) global efficiencies, and (d) local efficiencies for EC and EO states as a function of costs in the theta frequency band (4–7 Hz). Black asterisk indicated that the difference between EC and EO states was significant (paired *t*-test, *P* < 0.05). The *C*_*s*_ and local efficiencies of EC state were significantly higher than those of EO state in each cost. *L*_*s*_ and global efficiencies did not show significant difference between EC and EO states.

In the theta band (4–7 Hz), the mean cluster coefficient *C*_*s*_ of EC and EO states was shown as a function of costs in Figure 2(a). Within the whole costs (8%–20%), the *C*_*s*_ of EC state was significantly higher than that of EO state and the difference was consistent.  Figure 2(b) was the mean shortest path length *L*_*s*_, which decreased with the increase of costs, while the difference of each cost between EC and EO states was not significant at each cost. 

The efficiencies of EC and EO states in the theta band were shown in Figures 2(c) and 2(d), respectively, including global efficiencies and local efficiencies. Paired *t*-test showed no difference in global efficiencies between EC and EO states in the theta band, while the local efficiencies of EC state were higher than that of EO state at the whole costs. 

#### 3.1.2. Difference Nodes and Difference Connections

In order to further study the difference of network topology between EC and EO states, the crosstab test was used to determine difference nodes among all subjects in graphs. The node was determined as a difference node between EC and EO states when the node always showed significant difference at each cost.

**Figure 3:**
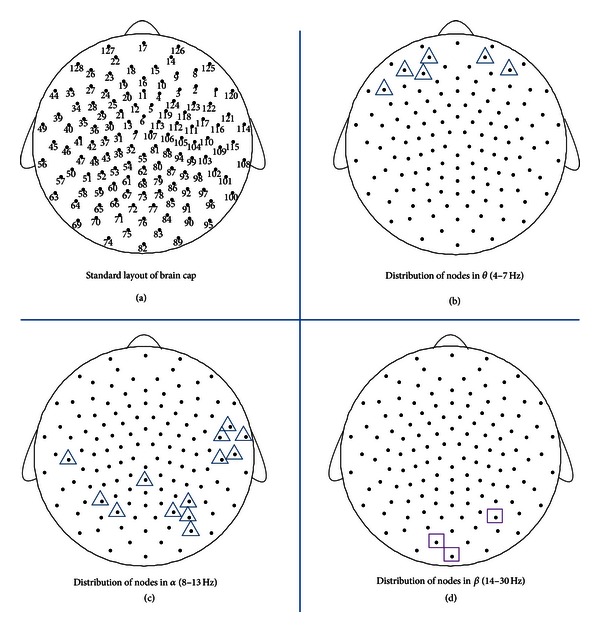
The distribution of difference nodes between EC and EO states in theta, alpha, and beta frequency bands (the crosstab test, *P* < 0.05). The difference node of EC state when the degree of a node in EC state was always significantly greater than that of EO state at each cost. The difference node of EO state when the degree of a node in EO state was always significantly greater than that of EC state at each cost. The triangle represented the difference node of EC state, and the rectangle represented difference node of EO state; (a) the standard layout of brain cap; (b) six difference nodes of EC state mainly located in the frontal area in the theta band (4–7 Hz); (c) thirteen difference nodes of EC state mainly located in the bilateral posterior area in the alpha band (8–13 Hz); and (d) three difference nodes of EO located in the occipital area in the beta band (14–30 Hz).

**Figure 4:**
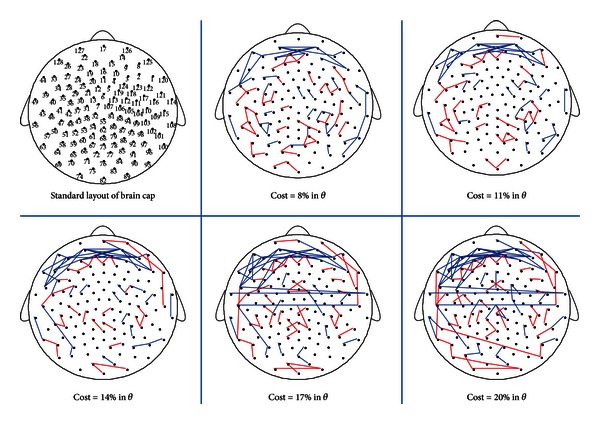
The difference connected graphs in the theta band (4–7 Hz) between EC and EO states in five costs. The first step is to construct the connected graphs of EC and EO states at each cost, which was composed of the edges which were significant among all subjects by the chi-square test (*P* < 0.05). The next step is to construct the difference connected graphs of EC and EO states at each cost. If only EC state had an edge in connected graph, the two nodes would be linked by a blue line. Otherwise, the two nodes were linked by a red line. The top left corner was the standard layout of the brain cap, with the positions of the electrodes indicated by small solid circles and numbered according to the 10–20 electrode placement system. The figure showed that the connections significantly reduced in the frontal area from EC to EO states in the theta band.

In the theta band, six significant difference nodes of EC state were distributed in the frontal area (See Figure 3(b)), showing that nodes of EC state had bigger degree than that of EO state.  Figure 4 showed difference connected graphs for difference connections between EC and EO state in range of all costs, which reveal that EC state had more connections than EO state in the frontal area at all costs. The distribution area of difference connections was in accordance with the area of difference nodes. All results suggested that the local activity of EC state was stronger than EO state in the frontal area in the theta band at resting state.

### 3.2. The Alpha Band

#### 3.2.1. Network Parameters

**Figure 5:**
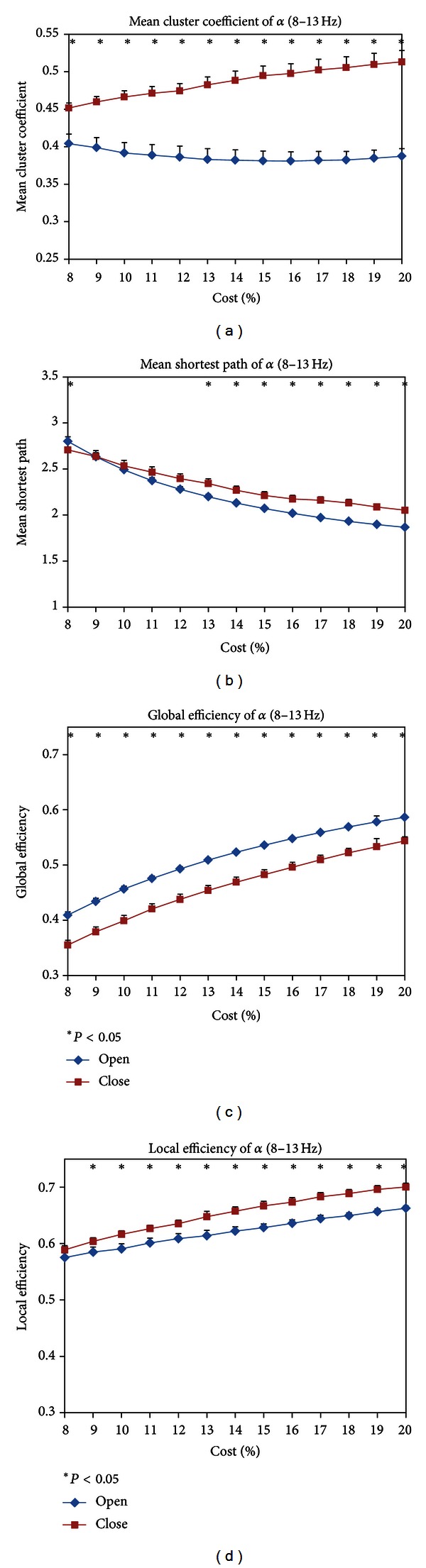
(a) mean cluster coefficient *C*_*s*_, (b) mean shortest path length *L*_*s*_, (c) global efficiencies, and (d) local efficiencies of EC and EO states as a function of costs in the alpha band (8–13 Hz). Black asterisk indicated that the difference between EC and EO states was significant at each cost (paired *t*-test, *P* < 0.05). *C*_*s*_, *L*_*s*_, and local efficiencies of EC state were significantly higher than EO state, but global efficiencies of EO state were significantly higher than those of EC state.

In the alpha band (8–13 Hz), the mean clustering coefficient *C*_*s*_ of EC state was significantly higher than that of EO state in the whole costs and the difference became larger as the costs increase in Figure 5(a).  Figure 5(b) illustrates the mean shortest path length *L*_*s*_ of EC and EO states as a function of costs. When the cost exceeded 13%, the *L*_*s*_ of EC was significant longer than that of EO state.

Figures 5(c) and 5(d) were the efficiencies of EO and EC states as a function of costs.  Figure 5(c) showed that EC state had lower global efficiencies than EO state. However, Figure 5(d) suggested that local efficiencies of EC state were higher than those of EO state when the cost exceeded 10%. 

#### 3.2.2. Difference Nodes and Difference Connections

**Figure 6:**
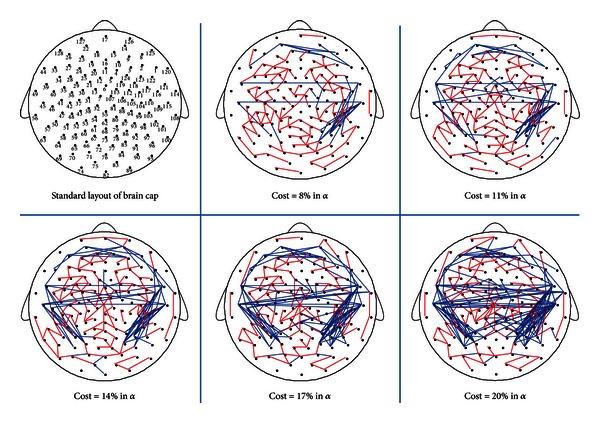
The difference connected graphs in the alpha band (8–13 Hz) between EC and EO states in five costs. The difference connected graph was constructed by the method in Figure 4. The blue line represented an edge only in connected graph of EC state, the red line represented an edge only in connected graph of EO state. The top left corner was the standard layout of the brain cap, with the positions of the electrodes indicated by small solid circles and numbered according to the 10–20 electrode placement system. The local connections significantly reduced in bilateral posterior areas from EC to EO states.

Thirteen difference nodes exhibited bilateral distribution mainly over the posterior area in the alpha band in Figure 3(c).  Figure 6 showed EC state had more connections than EO state which is located in the posterior area. The EC state had more local connections over the right posterior area when the cost was low (8% and 11%) and over the right and left posterior areas when the cost increased (14%, 17%, and 20%). Meanwhile, EC state had more and more long-range connections between the left and right posterior areas than that of EO state with increasing costs, while the difference connections of EO state scattered distribution throughout the whole brain in all costs. The above results showed that EC had stronger local activity in bilateral posterior areas and more communication between the left and right posterior areas than that of EO state in the alpha band at resting state.

### 3.3. The Beta Band 

#### 3.3.1. Network Parameters and Difference Nodes and Connections

**Figure 7:**
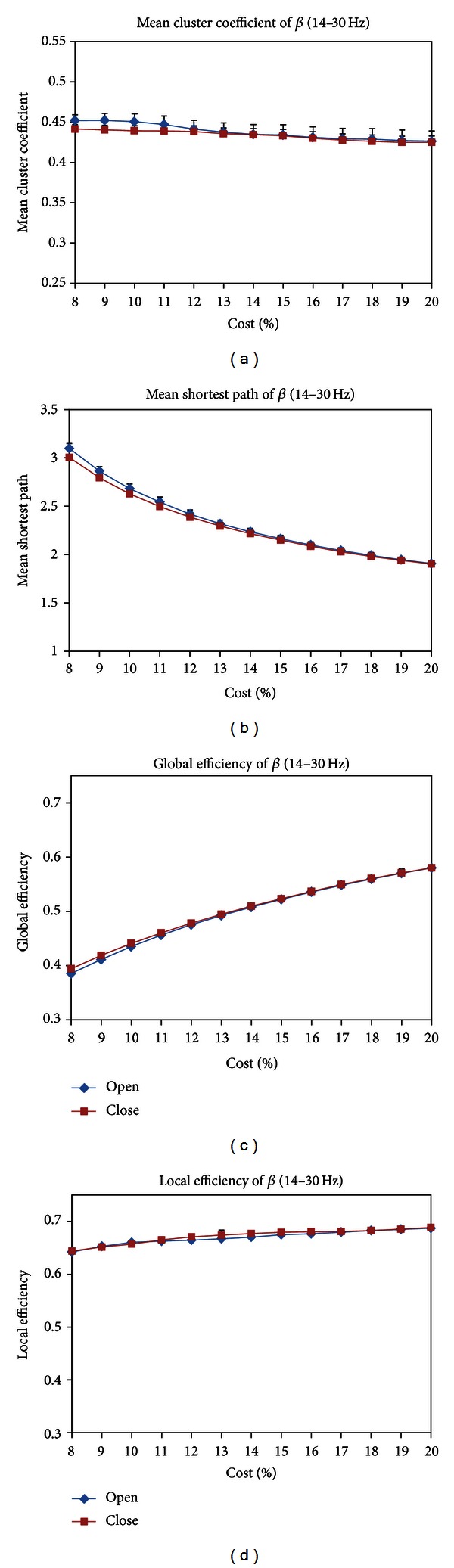
(a) mean cluster coefficient *C*_*s*_, (b) mean shortest path length *L*_*s*_, (c) global efficiencies, and (d) local efficiencies for EC and EO states as a function of costs in the beta band (14–30 Hz). Black asterisk indicated that the difference between EC and EO states was significant (paired *t*-test, *P* < 0.05). The *C*_*s*_, *L*_*s*_, global efficiencies, and local efficiencies did not show significant difference between EC and EO states in all costs.

**Figure 8:**
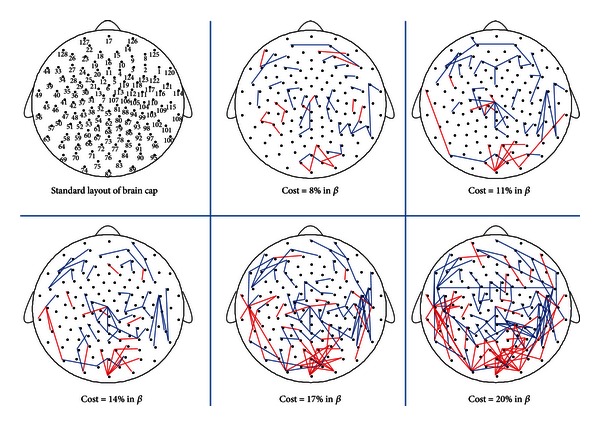
The difference connected graphs in the beta band (14–30 Hz) between EC and EO states in five costs. The difference connected graph was constructed by the method in Figure 4. The blue line represented an edge only in connected graph of EC state, the red line represented an edge only in connected graph of EO state. The top left corner was the standard layout of the brain cap, with the positions of the electrodes indicated by small solid circles and numbered according to the 10–20 electrode placement system. A few difference connections significantly reduced in the occipital area from EC to EO states.

In the beta band (14–30 Hz), the results of paired *t*-test showed no significant difference in the four topological parameters between EC and EO states in Figure 7. Three difference nodes of EO state located in the occipital area in Figure 3(d). The difference connections of EO state are mainly distributed in the occipital area in Figure 8. 

## 4. Discussion

The main goal of this paper was to address the differences of functional brain networks between EC and EO states. The results showed that EC state was characterized by a higher mean clustering coefficient *C*_*s*_ in both theta and alpha bands as well as a longer mean shortest path length *L*_*s*_ only in the alpha band. Local efficiencies were significantly reduced in both theta and alpha bands, but global efficiencies increased in the alpha band from EC to EO states. Moreover, after opening the eyes, both nodes and connections in the frontal area significantly decreased in the theta band and those of bilateral posterior areas also significantly decreased in the alpha band. But in the beta band, the topology of networks did not show much difference from EC to EO states.

### 4.1. Changes in Topological Parameters from EC to EO States in Theta Band

Chen et al. have reported that the local activity was suppressed and the power was drastically reduced in the frontal area from EC to EO state in the theta band [6, 7]. In consistence with these results, we found that the *C*_*s*_ and local efficiencies were significantly decreased from EC to EO states, and the nodes and connections significantly decreased from EC to EO states in frontal area in the theta band, which might be interpreted that visual input would suppress the connectivity of default mode network (DMN) after opening the eyes [3, 6, 9], resulting in a relatively spared local connectedness of DMN. The frontoparietal DMN has been suggested to be activated in eyes-closed resting state and suppressed after opening the eyes [31–33]. Decreased frontal theta rhythm has been found following a memory task [34], suggesting that there is a relationship between frontal theta and basic cognitive functions [34, 35]. The brain may prepare for cognitive activities after opening eyes, leading to a decrease of frontal theta rhythm from EC to EO states. In other words, the decrease of frontal theta in connectivity may play an inhibition role for the distraction of visual information in EO state [8, 13].

### 4.2. Changes in Topological Parameters from EC to EO States in Alpha Band

The *C*_*s*_, *L*_*s*_, and local efficiencies were significantly decreased from EC to EO states, suggesting different processing via oscillations in the two states. Zou et al. also indicated that the alpha rhythm had the largest amplitude in relaxed EC or a waken state [36]. These results were in line with other studies that the activity of the alpha would be restrained due to extrinsic visual stimulus and information processing in EO state [7, 8, 10]. In addition, we found the topological features of nodes and connections significantly reduced in bilateral posterior area from EC to EO states which would agree with previous studies that the alpha power mainly was distributed in bilateral posterior areas along and significantly reduced from EC to EO states, described as “alpha desynchronization” [6, 7, 9]. The alpha desynchronization to visual input has been considered to facilitate visual perception or information processing [9, 22]. Resting state fMRI data also have proved that the variations of alpha rhythm were associated with bilateral thalamic nuclei and visual cortex which modulated some basic cognitive function. Meanwhile, unlike other parameters, the global efficiencies were significantly increased inversely from EC to EO states, which may suggest that interactions among interconnected regions became more effective when we open the eyes [9, 37, 38]. Previous studies show that the shorter the path length, the higher the global efficiencies, which would be more conducive for promoting information exchanging and information processing [9, 37, 38]. Otherwise, long-range connections decreased between the left and right posterior areas from EC to EO states, which suggested that EO state had less communication between the two hemispheres and some brain functions may be suppressed.

### 4.3. The Small Worldness of Functional Brain Network between EC and EO States

**Figure 9:**
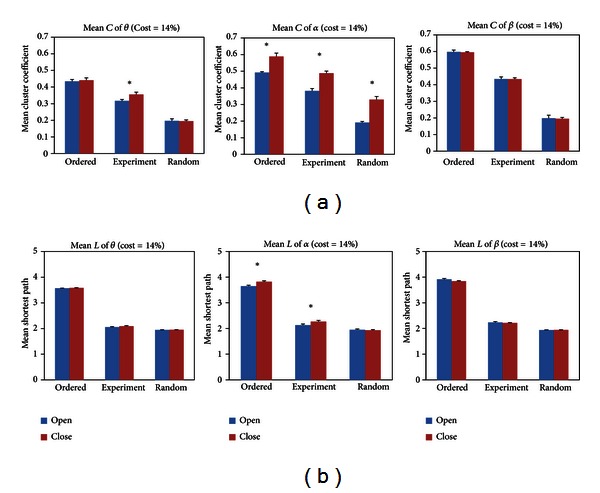
The comparisons of mean cluster coefficient *C*_*s*_ (the first row) and mean shortest path length *L*_*s*_ (the second row) among ordered, experiment, and random networks when the cost was 14%. Error bars corresponded to standard error of the mean. Black asterisk indicated that the difference between EC and EO states was significant (paired *t*-test, *P* < 0.05). The random and ordered networks were constructed with preserving the degree sequences of corresponding EC and EO networks. The *C*_*s*_ of EC and EO states were intermediates between that of ordered and random networks. However, the *L*_*s*_ of EC and EO states were lower than the *L*_*s*_ of ordered networks and close to (but not smaller than) the *L*_*s*_ of random networks.

Previous studies have shown that the brain network was a small-world network [20, 29, 39]. To further diagnose small worldness, the *C*_*s*_ and the *L*_*s*_ served as the main parameters to compare functional networks of EC and EO states with the ordered and random networks preserving the degree sequences of their experimental counterparts in each frequency band when the cost was 14% in Figure 9. The results showed that the *C*_*s*_ and the *L*_*s*_ of EC and EO states were intermediate between order and random networks and also suggested that the resting state networks had small-world features. To further analyze the difference of small-world features between EC and EO states, the *C*_*s*_ and the *L*_*s*_ were expressed as ratios of the *C*_*s*_ and *L*_*s*_ of random graphs in Table 1. The table showed that the small-world features reduced in the theta band but slightly increased in the alpha band from EC to EO states. The decrease of small-world features in the theta band may be due to the external visual input which induces a decrease of DMN activity [32, 33]. Besides, the increase of small-world features in the alpha band may be the alpha desynchronization after opening the eyes, which facilitates effective information communication [36–38]. Meanwhile, this study also illustrated small-world features of functional brain networks at resting state.

### 4.4. The Application of SL and Graph Theoretical Analysis in Neuroscience

The relationship between brain structure and function is a popular research area in the modern network theory, especially in synchronization dynamics and network topological features [11, 14, 15]. A growing body of evidence suggests that the brain is a nonlinear dynamic system, and coupled neurons synchronously produce brain signals [5, 16, 18]. The studies of synchronization dynamics are not thorough enough in the human brain and the synchronization analytical approach mainly concentrated on linear and nonlinear analysis [14, 15]. Nonlinear synchronization algorithms have been widely applied to analyze the human brain, especially phase synchronization and synchronization likelihood. Because of the nonstationary properties of EEG, many synchronization algorithms are not very suitable to EEG data [11]. The SL is sensitive to the nonstationary signal; so it has been applied to human multichannel EEG and MEG data. An important application field of the SL is neuronal diseases, such as Epilepsy, Alzheimer's disease [14, 15], and advanced brain functions, such as attention, perception, and memory [11, 40]. Using the method of the SL, studies have found some differences between neurologic patients and normal subjects. Moreover, other studies further suggested that synchronism activities of Alzheimer's and Epilepsy patients may play an important role in the information integration among different brain regions in the gamma band [40, 41]. The recent studies suggested that synchronous oscillations also had a vital role for higher cognitive function [11, 13, 41].

In neuroscience, some studies have shown the human brain could be modeled as a complex network and had the “small-world features” which are a relatively high clustering coefficient and a short path length at the level of anatomical connectivity as well as functional connectivity [4, 13, 28]. Simultaneously, the small-world framework was deemed to be an ideal situation, which was bound up with the most economic wiring cost, the information transmission, and the best balance between global information integration and local information processing [13, 17, 18]. Graph theory has been widely applied to study functional connectivity, neural anatomical, and connectivity networks models based upon EEG, MEG, and fMRI for many years [14, 16, 17, 30]. Stephan also indicated that theory graph could be well applied to analyze anatomical and functional connectivity in neuroscience [4, 42]. We believe the graph theory will have a broader application in the other fields.

### 4.5. The Limitation of Application in Neuroscience

The study has two main limitations. First, the nodes of network which were the scalp electrodes are not really anatomical positions. Because of the low spatial resolution in EEG, the topological findings must be interpreted with extreme caution. The CSD was used for compensating spatial resolution of EEG channels in this study. Second, while the SL has a wide application in the electrophysiological data (such as EEG, MEG), it is rarely used to analyze the neuroimaging data (such as fMRI, PET) due to the lack of enough time points for the SL calculation.

## 5. Conclusion

In this paper, we mainly discussed the difference of the functional brain networks between EC and EO states. The SL was applied to construct functional brain network and graph theory was applied to analyze the characteristics of functional connected graphs. The results showed that some topological parameters between EC and EO states had significant difference. Meanwhile, we found that the connectivity of frontal theta and posterior alpha significantly decreased from EC to EO states. In conclusion, these results suggest that the combination of the SL and graph theory would be a very useful tool to explore underlying mechanisms of brain.

## Figures and Tables

**Table 1:** Small-world feature of EC and EO states (mean cluster coefficient *C*_*s*_ and mean shortest path length *L*_*s*_ were expressed as ratios of corresponding *C*_*s*_ and *L*_*s*_ of random network).

Frequency range	State	*γ* = *C*_exp⁡_/*C*_ran_	*λ* = *L*_exp⁡_/*L*_ran_	*σ* = *γ*/*λ*
Theta (4–7 Hz)	EO	1.61	1.06	1.52
	EC	1.82	1.07	1.70
Alpha (8–13 Hz)	EO	2.01	1.09	1.83
	EC	1.48	1.18	1.26
Beta (14–30 Hz)	EO	2.19	1.15	1.91
	EC	2.23	1.14	1.95

## Appendix

### A. Mathematical Detail of Network Measures

#### A.1. The Degree and Degree Distribution

The number of existed edges between the adjacent nodes of a node is defined as the degree *K* of the node. For instance, the degree of node A is two in Figure 1(b). Many other fundamental parameters are related with the degree of a graph. The degrees of all nodes form a degree distribution of a graph and the mean degree of a graph is to average all nodes degrees [17, 18, 25]. However, because the threshold was based on the cost which is in accordance with the degree of a graph, the degree of EC and EO states did not show difference and would not be discussed in the paper.

#### A.2. The Clustering Coefficient and Mean Clustering Coefficient

The clustering coefficient is used to measure clustered degree among nodes and an index of local structure in a graph. The clustering coefficient of a node is usually defined as the proportion of the number of existed edges between the nearest neighbor of the node and the maximum number of possible edges. For example, the clustering coefficient of node C is 1 in Figure 1(b). It ranges between 0 and 1. The mean clustering coefficient *C* of all nodes was calculated by averaging the *C*_*s*_ of all nodes in a graph. In this study, we compared the mean *C*_*s*_ between EC and EO states. The formulas of the clustering coefficient *C* and the mean *C*, respectively, are defined as follows [16, 25]:

(A.1) $$\begin{array}{c} C_{i}=\frac{e_{j}}{C_{n}^{2}}=\frac{2e_{j}}{k_{j}\left(k_{j}-1\right)}, \end{array}$$

(A.2) $$\begin{array}{c} C_{\text{mean}}=\frac{1}{N}\sum_{j=1}^{N}C_{j}, \end{array}$$

where *e*_*j*_ is the number of existed edges between adjacent nodes of node *i* and *k*_*j*_(*k*_*j*_ − 1) is the maximum possible number of edges among neighbours in formula (A.1). *N* is the number of nodes in a graph and *C*_mean_ is the mean clustering coefficient in a graph.

#### A.3. The Shortest Path Length and Mean Shortest Path Length

The shortest path length between two nodes is the minimal number of edges that must be travelled to go from one node to the other [17, 18, 25]. For example, the path length between node A and node D is 2 in Figure 1(b). The average of all the shortest path lengths between all possible pairs of nodes is called the mean shortest path length *L* of a graph. The formula of *L* in a graph is defined as follows:

(A.3) $$\begin{array}{c} L_{i,j}=\frac{1}{N\left(N-1\right)}\sum_{i,j\in N,i\neq j}d_{i,j}, \end{array}$$

where the distance *d*_*i*,*j*_ between the two nodes *i* and *j* is the minimal number of edges that has to be travelled to go from node *i* to node*j*. *L*_*i*,*j*_ is the average of all nodes' path length *d*_*i*,*j*_.

#### A.4. The Global Efficiency *E*_global_ and Local Efficiency *E*_local_

Latora and Marchiori present the definitions of the global and local efficiency of a graph. The global efficiency is measured by the inverse of the harmonic mean of the minimum path length between each pair of nodes [17–19]. If one node is separate with the other nodes, the path length is considered to be infinite and the efficiency is zero. Meanwhile, the global efficiency can also reflect the level of parallel information transfer of a network. The equation of global efficiency is [18, 19]

(A.4) $$\begin{array}{c} E_{\text{global}}=\frac{1}{N\left(N-1\right)}\sum_{i,j\in N,i\neq j}\frac{1}{d_{i,j}}. \end{array}$$

The mean of the efficiencies of all subgraphs *G*_*i*_ of neighbours of each node of the graph is defined as the local efficiency of a graph [18, 19], and the average local efficiency *E*_local_ is given by

(A.5) $$\begin{array}{c} E_{\text{local}}=\frac{1}{N}\sum_{i\in N}E\left(G_{i}\right), \end{array}$$

where *E*(*G*_*i*_) is the efficiency of subgraph *G*_*i*_; the local efficiency is also understood as a measure of the fault tolerance of the network, indicating how well each subgraph exchanges information when the index node is eliminated [18, 19].
